# Fabrication of Wear-Resistant and Anti-Reflection Surfaces Based on Armor-Protected Nanocone Structures

**DOI:** 10.3390/mi17030360

**Published:** 2026-03-15

**Authors:** Haoyu Tian, Jianxun Chen, Jiaheng Bi, Haotian Guo, Cheng Lei, Ruirui Li

**Affiliations:** State Key Laboratory of Extreme Environment Optoelectronic Dynamic Measurement Technology and Instrument, North University of China, Taiyuan 030051, China

**Keywords:** antireflection, nanocones, protective armor, wear resistance, RIE

## Abstract

Antireflection surfaces play an indispensable role in modern optics, with extensive applications covering optical windows and other precision optical components. The fabrication of anti-reflection surfaces frequently relies on micro/nano-structuring technologies. However, the fabricated micro/nanostructures typically experience performance degradation in transmission enhancement caused by abrasion during operation. To address this problem, we designed and fabricated a double-sided nanocone structure shielded by a protective armor layer. This armor layer efficiently prevents surface mechanical wear and preserves the nanocone structures, leading to almost constant transmittance of the anti-reflection surface even after abrasion. The anti-reflection surface was fabricated by first patterning a square grid armor on one side of fused silica via photolithography, followed by the preparation of an etching mask and nanocone structures using reactive ion etching (RIE). Nanocones were then fabricated on the opposite side of the substrate, finally forming the double-sided nanocone structure. The fabricated armor-protected double-sided nanocone structure exhibited an increase in the average transmittance from 93.43% to 98.31% within the wavelength range of 800–1200 nm. After abrasion testing under 10 MPa pressure, the nanocones under the protective armor showed almost no damage, and the average transmittance remained at approximately 97.85%, demonstrating the outstanding mechanical robustness of the proposed design.

## 1. Introduction

Recent investigations have verified that broadband anti-reflection techniques can substantially elevate the performance of a wide spectrum of optical components, including optical windows, laser systems, sensing and detection facilities, and medical optical apparatus [1,2,3,4,5]. This enhancement is attributed to the mitigation of surface reflection, which effectively increases the total optical transmittance by minimizing surface light reflection. Two main approaches can achieve broadband antireflection effectively. One relies on optical thin-film deposition, while the other uses subwavelength structures, such as nanocone structures. For thin-film deposition, researchers have developed various material systems, including conventional fluorides [6], sulfides [7], and oxides [8], as well as novel materials such as germanium carbide films [9], nanoporous structures [10], and noble metal nanofilms. Although thin-film coatings can deliver favorable broadband antireflection performance, they generally suffer from inherent limitations, including poor adhesion, insufficient stability, low laser-induced damage threshold, and thermal expansion mismatch [11]. In contrast, nanocone structures are fabricated directly from the substrate itself. Since no additional external materials are involved, they exhibit superior mechanical stability and durability compared to optical thin films [12]. However, as the antireflection function of nanocone structures depends on the light refraction at the cone tips, the antireflection effect will be significantly compromised once the cone tips are damaged.

Nanocone structures also have a wide variety of fabrication methods, most of which are based on top-down photolithography or etching techniques [13], such as monolayer colloidal crystal etching [14,15], electron-beam etching [16,17], interference lithography [18,19,20], and nanoimprint lithography [21,22,23]. In addition, laser processing has also been employed for the fabrication of nanocone structures [24,25]. However, in comparison with etching techniques, laser processing is a serial manufacturing approach with relatively low processing efficiency. One of the most critical limitations of antireflection structures for transmission enhancement is their poor wear resistance [26]. Xu et al. prepared a trilayer hydrophobic antireflective coating based on the sol–gel method, achieving an average transmittance of 97.77% and a transmittance decrease of 1.63% after abrasion testing [27]. Zhang et al. fabricated a coating by modifying hollow SiO_2_ nanospheres with methyltriethoxysilane (MTES) and tetraethyl orthosilicate [28]. The coating exhibited an average transmittance of 95.61% and a transmittance decrease of 0.6% after abrasion testing. However, the uniformity of such wear-resistant nanostructured coatings must be carefully ensured in practical applications; otherwise, the optical transmittance will be compromised. Wang et al. fabricated an armor-protected wear-resistant structure on fused silica substrates via embossing technology [29]. The nanostructures were formed on the recessed oblique surfaces of fused silica, and their transmittance was only approximately 94.5%, failing to achieve broadband supertransmissivity. Xu et al. prepared armored nanocone structures through selectively laser-doping-enhanced plasma etching (SDEM) [26]. The double-sided structured fused silica exhibited an average transmittance of around 97% in the 0.4–1.2 μm wavelength range, with no significant decrease in transmittance after 150 cycles of abrasion. However, compared with plasma etching technology, laser processing is a serial manufacturing method, leading to relatively low processing efficiency in large-area fabrication.

In this study, a fabrication method for double-sided nanocone structures is proposed, which enables parallel large-area processing while achieving both high wear resistance and high transmittance. A micro-scale grid armor structure is prepared to improve the wear resistance of the substrate, followed by the construction of double-sided nanocone structures to reduce surface reflection and enhance transmittance. The micro-scale grid armor structure is fabricated on a fused silica substrate via photolithography and RIE. After the formation of the protective armor, a nanofiber etching mask is created by treating the photoresist with oxygen plasma, and nanocone structures are subsequently formed via RIE. Upon completing the nanocone etching on the opposite side, the double-sided nanocone structure with high wear resistance and high transmittance is finally obtained. Then, the transmission properties of nanocone structures with different dimensions are simulated and evaluated using the finite-difference time-domain (FDTD) method. Meanwhile, finite element analysis is employed to investigate the bottom stress distribution of armor sidewalls with different geometries, aiming to achieve optimal wear resistance. This work integrates the fabrication of nanocone with wear-resistant armor structures, providing a reliable technical strategy for realizing antireflection performance. It helps promote the practical application of highly transparent optical windows in wear-resistant scenarios.

## 2. Materials and Methods

### 2.1. Materials

Fused silica substrate (Corning 7980) was purchased from Research Materials Microtechnology Co., Ltd., Suzhou, China. Polyimide (PI) photoresist was purchased from Beijing POME Science and Technology Co., Ltd. (Beijing, China).

### 2.2. Fabrication of Protective Armor

An AZ-6130 photoresist layer was spin-coated onto the cleaned fused silica substrate and soft baked at 100 °C for 60 s. After exposure using the EVG610 photolithography machine, the sample was hard-baked at 120 °C for 100 s. The sample was then developed using TMAH, washed with deionized water, and dried with nitrogen. CHF_3_ and Ar plasma were used to etch the fused silica substrate, forming the protective armor structure. The RF power for plasma etching was set to 210 W, with a chamber pressure of 2.7 Pa. The flow rates of CHF_3_ and Ar were 70 sccm and 5 sccm, respectively. After etching, the sample was cleaned sequentially with acetone, ethanol, and deionized water to remove any residual photoresist. The protective armor structure was created using the RIE-10NR (SAMCO International, Kyoto, Japan) etching system.

### 2.3. Fabrication of Nanofiber Mask

The PI photoresist layer was spin-coated onto the cleaned fused silica substrate. The PI photoresist was exposed to O_2_ plasma, creating the nanofiber mask. The RF power was set to 210 W, with a chamber pressure of 5 Pa and an O_2_ flow rate of 125 sccm. In this experiment, the spin rate varied (2500 rpm, 3500 rpm, 4500 rpm, and 6000 rpm) to explore the relationship between spin speed and the etching time required to create the mask. Nanofiber structures were fabricated using the RIE-10NR (SAMCO International, Kyoto, Japan) etching system.

### 2.4. Fabrication of Nanocone Structure

Nanocone structures were prepared on fused silica substrates via RIE with nanofiber masks. SF_6_, CHF_3_, and He were introduced at flow rates of 5.5, 35, and 160 sccm, respectively. With a chamber pressure of 245 Pa and RF power of 210 W, the etching time was adjusted from 1 to 4 min to achieve different nanocone structure morphologies. After etching, residual nanofiber masks were stripped using buffered oxide etch (BOE) solution, achieving the well-defined nanocone structures. The nanostructures were fabricated using the RIE-10NR (SAMCO International, Kyoto, Japan) etching system.

### 2.5. Fabrication of Double-Sided Nanocone Structures with Protective Armor

After sequentially fabricating the protective armor and nanocone structure on one side of the fused silica substrate, nanocone structures were prepared on the opposite side. The sample was then cleaned using BOE solution, acetone, ethanol, and deionized water in sequence, finally achieving a double-sided nanocone structure with protective armor.

### 2.6. Wear Test

The double-sided nanocone structure with protective armor was subjected to wear testing using the friction and wear tester (MFT-2000, RTEC). The applied normal force was 3.92 N, resulting in a normal pressure of 10 MPa for a line width of 2.5 μm and a line length of 1000 μm. The tester recorded a force of 1.44 N in the wear direction, corresponding to a shear stress of 3.66 MPa. Friction testing was conducted using an acrylonitrile butadiene styrene (ABS) plastic rod (diameter 10 mm) for 100 cycles. Following the wear test, the sample was cleaned via ultrasonic washing with ethanol and deionized water.

### 2.7. Characterization

The morphology of the protective armor, nanofibers, and nanocones were characterized using a scanning electron microscope (SEM) (Gemini SEM500, Zeiss Oberkochen, Germany). Spectrum data of transmittance were measured with a spectrometer (LAMBDA 750S, PerkinElmer, Waltham, MA, USA).

### 2.8. Simulation Methods

The transmittance simulation was carried out using the Ansys Lumerial 2020 R2 software for the nanocone model. The nanocone was set to be made of silicon oxide. Periodic boundary conditions were applied in the X-direction, while perfectly matched layer boundary conditions were employed in the Z-direction. A plane-wave light source with a specified bandwidth was incident along the Z-direction, which is a transverse electromagnetic wave with constant amplitude on the plane perpendicular to the propagation direction. The incident angle was set to 90°, and a detector was placed beneath the nanocone model to monitor the transmittance. The mesh refinement level was set to 7, and the simulation domain fully enclosed the nanocone model. For the transmittance simulation with different bottom diameters of the nanocone, the height was set to 480 nm, the duty cycle to 1, and the wavelength range to 400–1300 nm. The bottom diameter was set to 272 nm, 282 nm, 376 nm, 386 nm, 481 nm, 491 nm, 50 nm, 100 nm, 150 nm, 200 nm, and 250 nm respectively, and the corresponding transmittance values were obtained through simulation. For the transmittance simulation with different duty cycles, the height was set to 480 nm, the bottom diameter to 200 nm, and the wavelength range to 800–1200 nm. The transmittance variations were simulated at duty cycles of 0.5, 0.6, 0.7, 0.8, 0.9, and 1. For the transmittance simulation with different nanocone heights, the duty cycle was set to 1, the bottom diameter to 200 nm, and the wavelength range to 400–1300 nm. The transmittance variation was simulated with the height scanned from 0 to 700 nm.

The stress distribution simulation of the protective armor was performed using Ansys workbench 2022 R1. The length of the protective armor was set to 1000 μm, the width to 2.5 μm, and the material to fused silica, with a mesh resolution of 7. A horizontal shear stress of 3.66 MPa and a vertical pressure of 10 MPa were applied, and the bottom surface of the structure was set as a fixed support. The stress distributions were simulated and calculated for three protective armor shapes (regular hexagon, square, and equilateral triangle) and three sidewall inclination angles (75°, 90°, and 105°).

## 3. Design and Simulation

### 3.1. The Wear-Resistant Mechanism of Armor-Protected Nanocone Structures

**Figure 1:**
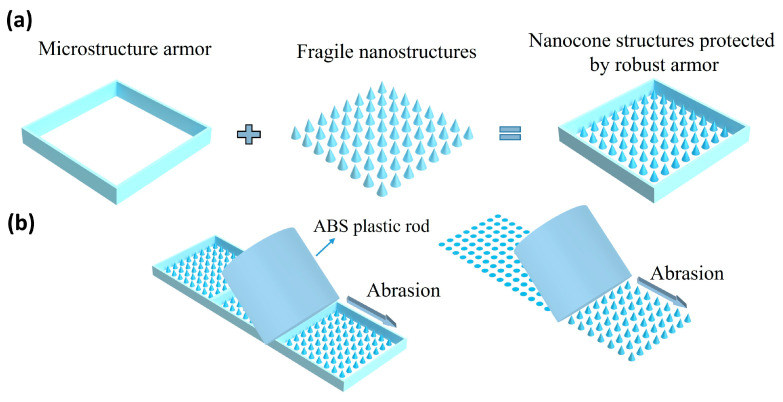
(**a**) Schematic diagram of the composition of armor-protected nanocone structures. (**b**) The wear resistance process of armor-protected nanocone structures.

Figure 1a shows the schematic design of the highly wear-resistant antireflection surface. The structure consists of a nanocone structure and a micro-scale protective armor, in which the micro-armor serves as the key component. It is composed of interconnected frameworks with multiple “pockets” inside, which are specifically designed to accommodate the nanocone structures with relatively poor mechanical robustness. Acting as a “protective framework” for the antireflection surface, the armor can effectively block abrasion or impact from objects larger than the pocket size, thus preventing damage to the nanocones during service. This protection mechanism ensures that the nanocones maintain their tip morphology, thereby prolonging their service life. In addition, the monolithic structure formed by the interconnected frameworks not only shields the nanocones, but also reinforces the mechanical stability of the entire surface, making the structure resistant to fracture under external pressure or impact. As shown in Figure 1b, the protective armor prevents the nanocone structure from wear damage. In the absence of this protective layer, abrasive structures would directly scrape the nanocones during relative motion, leading to a rapid degradation of optical performance. With the protective armor incorporated, its feature size is sufficiently small to block abrasive objects larger than its openings, such that these forces cannot reach the nanocones underneath, thereby reliably shielding the structure from mechanical damage.

### 3.2. Design of Nanocone Structure

**Figure 2:**
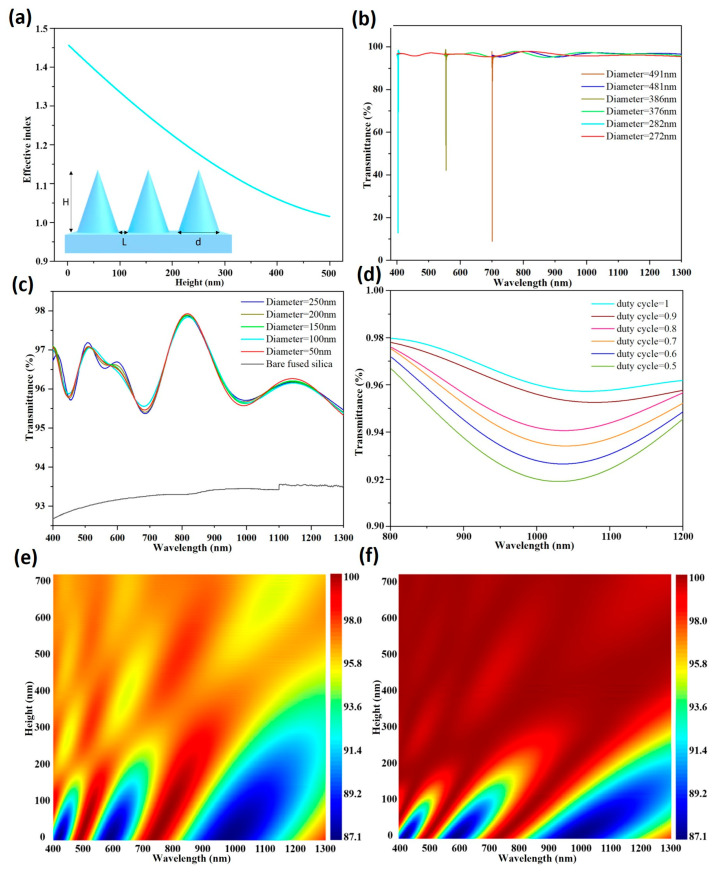
(**a**) Simulation results of the effective refractive index as a function of nanocone height (inset: nanocone structural parameters). Simulated transmittance for various (**b**,**c**) nanocone diameters and (**d**) duty cycles. Simulated scanning transmittance of (**e**) single-sided and (**f**) double-sided nanocone structures for various heights.

The nanocone structure primarily achieves broadband antireflection by suppressing Fresnel reflection at the boundary between media with differing refractive indices, and the underlying physical principle is based on the effective medium theory (EMT) for subwavelength nanostructures. EMT is a classic theoretical model for describing the optical response of composite materials with structural features much smaller than the incident light wavelength The effective refractive index of the nanocone structure is expressed as follows:

(1) $$n_{e}^{2}=n_{a}^{2}\cdot f+n_{s}^{2}(1-f)$$

where $n_{e}$ represents the effective refractive index, $n_{a}$ denotes the refractive index of air, $n_{s}$ refers to the refractive index of the fused silica substrate, and f signifies the volume fraction of air in the micro-nanostructure region. Based on this relationship, the specific value of the effective refractive index can be calculated. It can be derived from the formula that the gradual gradient variation of the refractive index at the interface is mainly caused by the change in the air volume fraction between the nanostructures. During incidence, the incident light first encounters air with a refractive index close to 1. As it propagates downward along the nanocones, the effective refractive index increases continuously and eventually approaches the refractive index of the substrate. This continuous variation in refractive index eliminates abrupt index discontinuities, which can significantly reduce surface reflection and thus achieve excellent antireflection performance. Figure 2a numerically demonstrates that the effective refractive index increases smoothly from the tips of the nanocones toward the base. The inset of Figure 2a displays the key structural parameters of the nanocone architecture. The height H, diameter d, and duty cycle are critical factors governing its optical properties. The duty cycle (the ratio of the area occupied by the nanocone structure to the total area) quantifies the compact packing degree of the nanocones. This ratio is equal to d/(L + d), where L is the minimum linear distance between the bottoms of the nanocones. To more comprehensively investigate the influence of these parameters on light transmittance, a series of FDTD simulations were performed.

When investigating the effect of nanocone diameter on transmittance, the height was fixed at 480 nm. We simulated the transmittance of nanocones with diameters of 272 nm and 282 nm over the 400–1300 nm wavelength range, those with diameters of 376 nm and 386 nm over 550–1300 nm, and those with diameters of 481 nm and 491 nm over 700–1300 nm. The wavelengths of 400 nm, 550 nm, and 700 nm served as the minimum incident wavelengths for the three sets of simulations, corresponding to fused silica refractive indices of 1.47, 1.46, and 1.455, respectively. The simulation results are presented in Figure 2b. For diameters slightly smaller than the ratio of the shortest wavelength to the refractive index (272 nm, 386 nm, 481 nm), the transmittance varies smoothly with wavelength without any abrupt drops. In contrast, for diameters slightly larger than this ratio (282 nm, 396 nm, 491 nm), a significant decrease in transmittance is observed at certain short wavelengths. This is likely caused by strong surface scattering induced when the diameter exceeds λ/$n_{s}$. In addition, we simulated nanocones with diameters of 250 nm, 200 nm, 150 nm, 100 nm, and 50 nm across the 400–1300 nm wavelength range, as shown in Figure 2c. The results showed only minor variations in transmittance, further suggesting that the diameter should be below λ/$n_{s}$. For instance, at a wavelength of 800 nm, the refractive index of Corning 7980 was 1.452, meaning that the diameter should be less than 550.96 nm to achieve optimal transmittance in the 800–1200 nm range. The duty cycle is also a critical factor in determining the optical performance of nanocone structures. As shown in Figure 2d, we conducted FDTD simulations with the cone height set at 480 nm and diameter at 200 nm, calculating the transmittance at duty cycles of 0.5, 0.6, 0.7, 0.8, 0.9, and 1. The results demonstrated that higher duty cycles led to better transmittance. The optimal performance occurred at a duty cycle of 1. The duty cycle in the simulation represents only the nanocone structure without the protective frame, rather than the entire patterned surface including the frame.

With the diameter still fixed at 200 nm, we analyzed the influence of height on the transmittance of single-sided nanocone structures, with the structure height ranging from 0 to 700 nm (see Figure 2e). The results showed that the transmittance increased with the increase in structure height. When the height exceeded 470 nm, the structure exhibited optimal performance in the 400–1300 nm band. Taller nanocones contributed to a more gradual gradient of the effective refractive index, thereby reducing reflection losses. We also performed simulations on double-sided nanocone structures over the same height range (Figure 2f). Overall, the double-sided structure presented higher transmittance than the single-sided one. In the 800–1200 nm band, a height slightly above 300 nm was sufficient to achieve peak performance. This performance improvement was attributed to the deployment of nanocone structures on both surfaces of the structure, which further suppressed Fresnel reflections and optimized optical performance. The results indicate that the ideal nanocone structure should satisfy four criteria: the duty cycle should be as close to 1 as possible, the height is preferably above 300 nm, the diameter is preferably below 550.96 nm, and the nanocones should be fabricated on both sides of the substrate. The simulation model of the nanocones is a simplified standard model. All simulation results only reflect the trend of optical performance variation with structural parameters and provide theoretical guidance for experiments, rather than precise quantitative predictions of the actual structural dimensions.

### 3.3. Design of Protective Armor

The stability of the protective armor is a core consideration in its structural design. Only with excellent mechanical stability can the armor effectively shield the nanocone structures from harsh environments, prevent structural damage, and ensure that its antireflection function remains intact. We improved the structural stability by regulating the micron-scale morphology and sidewall inclination angle of the protective armor.

**Figure 3:**
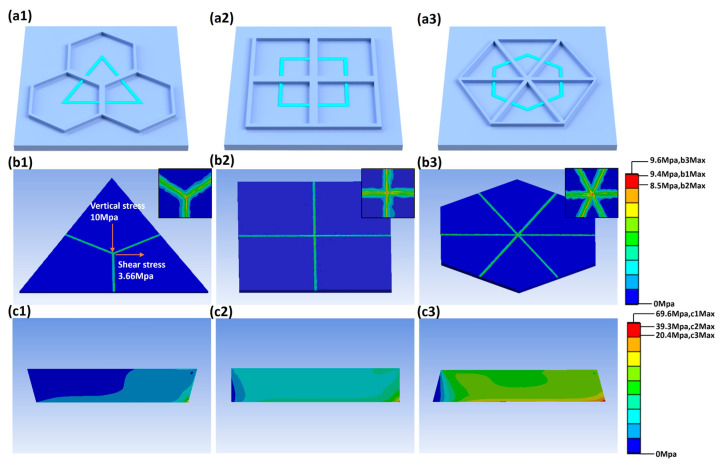
Schematics of basic stress-bearing units of (**a1**) hexagon unit (3 branches per node), (**a2**) square unit (4 branches per node), and (**a3**) triangle unit (6 branches per node). Finite element simulations of different armor shapes: (**b1**) hexagon unit, (**b2**) square unit, and (**b3**) triangle unit. Finite element simulations of different sidewall angles: (**c1**) 75°, (**c2**) 90°, (**c3**) 105°.

Regarding the armor morphology, we compared and analyzed three types of grid unit structures: regular hexagon, square, and equilateral triangle. The side length of all of these structures was set to 1000 μm and the width to 2.5 μm, and their basic stress-bearing units are shown in Figure 3(a1–a3). Each node of the regular hexagonal grid is connected to three branches, the square grid to four branches, and the triangular grid to six branches. Simulations were carried out under an applied shear stress of 3.66 MPa. The finite element analysis results are displayed in Figure 3(b1–b3). The maximum stress generally occurs near the nodes, and the enlarged stress distribution diagrams at the nodes for the three structures are shown in the upper right corner of Figure 3(b1–b3). The square unit exhibits the smallest maximum equivalent stress, followed by the regular hexagonal unit, while the equilateral triangular unit shows the largest maximum equivalent stress, indicating that the square design provides optimal structural stability. In addition, we performed simulation analyses of the equivalent stress at three different sidewall inclination angles (75°, 90°, and 105°) under identical loading conditions. The results shown in Figure 3(c1–c3) reveal that the equivalent stress of the structure is the lowest at an inclination angle of 105°. Compared with the 90° case, increasing the inclination angle by 15° reduces the stress to 1/1.96 of the original value. A larger inclination angle further decreases the stress, which corresponds to enhanced structural strength, but it also reduces the distribution area of nanocones, thereby affecting the transmittance. The stress reaches its maximum at an inclination angle of 75°; relative to the 90° structure, decreasing the angle by 15° increases the stress by a factor of 1.77. Moreover, the stress amplification becomes more significant as the angle decreases, which raises the risk of structural cracking and wear. Therefore, an obtuse angle is adopted for the armor sidewalls rather than a right angle or an acute angle. In actual fabrication, the protective armor with such obtuse sidewall angles can be relatively easily prepared using photolithography and RIE techniques.

**Figure 4:**
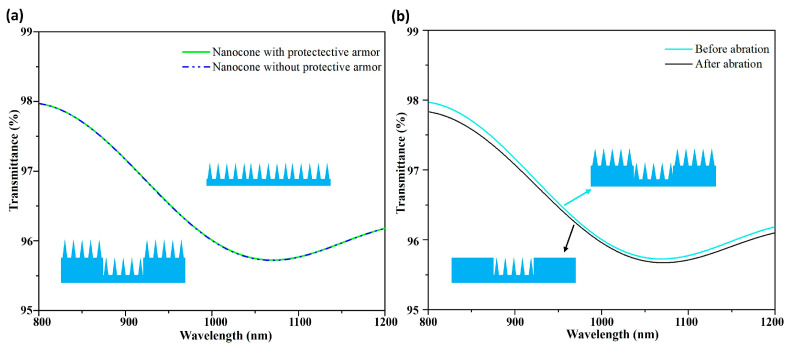
(**a**) Simulated total transmittance of nanocone structures with and without protective armor. (**b**) Simulated total transmittance of armor-protected nanocone structures before and after abrasion.

To investigate the influence of protective armor on transmittance, we performed simulation comparisons on the transmittance of nanocone structures with and without protective armor. The nanocone diameter, height, and duty cycle were set to 200 nm, 480 nm, and 1, respectively. As can be seen from Figure 4a, the protective armor has almost no effect on the transmittance of the nanocone structures. This is because nanocone structures are also fabricated on top of the protective armor during the preparation process (inset of Figure 4a), so the regions covered by protective armor exhibit the same transmittance. Therefore, the influence of protective armor on the transmittance of nanocone structures can be neglected. We further investigated the variation in transmittance after surface wear. The nanocones were set to have a diameter of 200 nm, a height of 480 nm, and a duty cycle of 1. Assuming that the nanocone structure on top of the protective armor was worn away, the proportion of nanocones located on the armor is very small, which means that only a slight change in the duty cycle of the overall nanocone structure is induced. The smaller the area of the protective armor, the fewer nanocone structures are damaged, resulting in a very small reduction in transmittance. Therefore, to balance structural protection and optical performance, we adopted a minimized design for the dimensions of the protective armor. This design can effectively resist most wear while minimizing its impact on the duty cycle of the structure.

Considering both application requirements and fabrication constraints, the final design adopted a protective armor with a length of 1000 μm and a width of 2.5 μm. At this size, the structural duty cycle decreases by only 0.5%, even when wear occurs. As shown in Figure 4b, FDTD simulations were performed to compare the optical performance at duty cycles of 1 and 99.5%. The results demonstrate that a 0.5% reduction in duty cycle causes only extremely slight variation in transmittance.

## 4. Fabrication and Results

**Figure 5:**
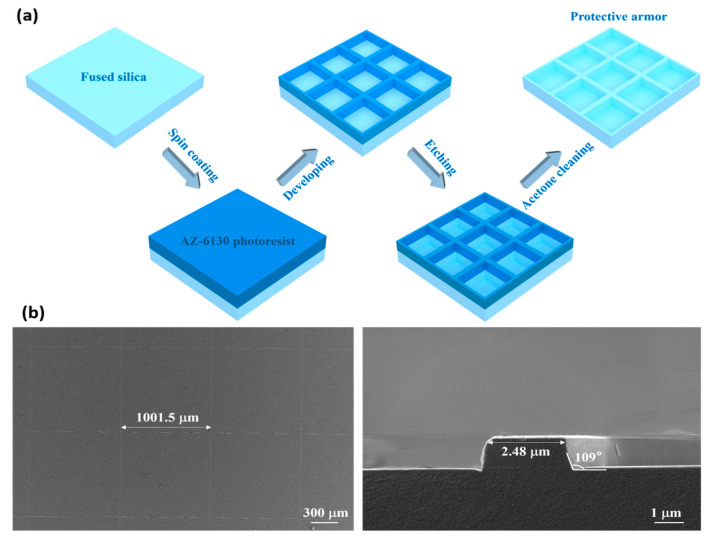
(**a**) Fabrication process of protective armor. (**b**) SEM images of protective armor.

The fabrication of armor-protected nanocone structures involves two processes: the preparation of the protective armor and the preparation of the nanocone structures. For the fabrication of protective armor, square protective armor structures were fabricated on the fused silica surface using photolithography and RIE techniques. The procedure included substrate cleaning, photoresist coating, pre-baking, lithography, development, and post-baking. Using the photoresist as a mask, the protective armor structures were etched using RIE. After etching, the residual photoresist was removed, yielding the square protective armor. The protective armor fabrication process is shown in Figure 5a, The protective frame exhibits a periodic structure with a cycle length of approximately 1000 μm. Figure 5b shows a SEM image of the protective armor. The armor has a square grid structure with a side length of approximately 1000 μm, which can block wear on the internal nanocone structures caused by external forces. With a linewidth of around 2.5 μm, this design minimizes the influence of the armor on the duty cycle of the nanocone structure and improves the optical transmittance. The sidewalls adopt an obtuse-angle design (~109°), which helps reduce the stress peak and enhance wear resistance.

**Figure 6:**
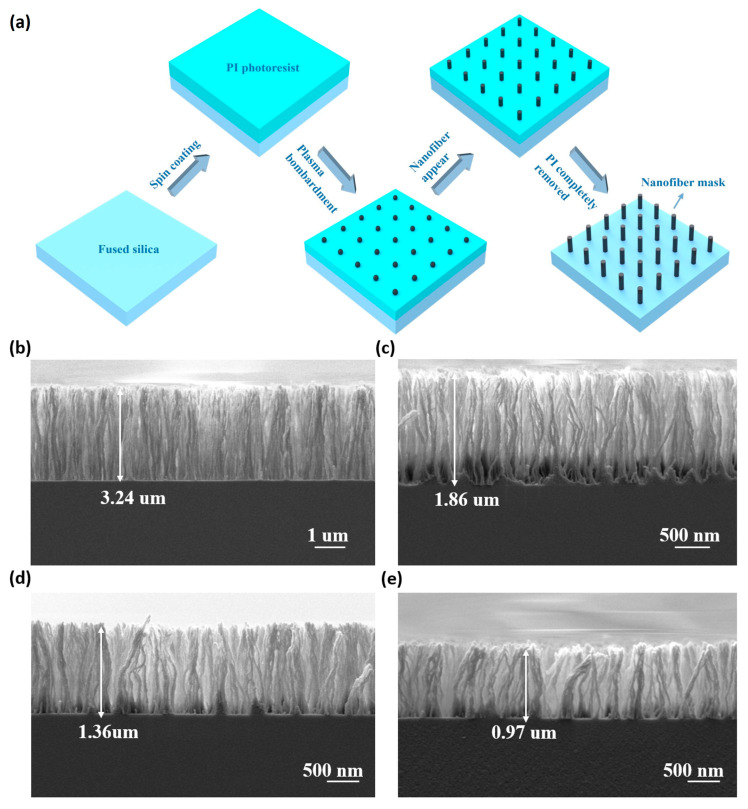
(**a**) Nanofiber mask fabrication process. (**b**–**e**) SEM images of nanofiber mask formed using spin-coating speeds of (**b**) 2500 rpm, (**c**) 3500 rpm, (**d**) 4500 rpm, and (**e**) 5500 rpm.

After fabricating the protective armor via photolithography and RIE, the next step is to prepare the nanofiber mask for the construction of the nanocone structure, whose process flow is illustrated in Figure 6a. A layer of PI photoresist is uniformly spin-coated onto the surface of the cleaned fused silica substrate, followed by oxygen plasma treatment. During the initial stage of oxygen plasma treatment on the PI layer, the outermost layer of PI decomposes into imide monomers and other low-molecular-weight compounds. As the plasma treatment progresses, these low-molecular-weight compounds are removed, leaving only the imide monomers on the surface of the PI layer. Meanwhile, under the action of the plasma, the organic components on the surface of the PI layer also decompose, exposing the silane coupling agents, which then immediately begin to form nanoparticle structures. Subsequently, during the high-energy dissociation phase of the plasma, the imide monomers remaining on the PI layer surface dissociate, generating ions, free electrons, neutral particles, and reactive (reaction) fragments. These particles and fragments then recombine to form a novel plasma polymer (also termed nanoresidues), which begins to exist in a crosslinked structure; at the same time, the nanoparticle structures undergo a polymerization reaction with these dissociation products, eventually forming fibrous cross-linked polymer structures. As the oxygen plasma treatment continues, the PI layer progressively thins and is gradually removed: on the one hand, more nanoresidues are synthesized and grafted onto the previously formed cotton-like structure, ultimately forming vertical nanowires; on the other hand, the height and diameter of the fibrous cross-linked structures increase continuously. Once the PI layer is completely removed, the nanofiber mask structure is obtained [30]. We conducted a comparative analysis of the nanofiber structures formed at different spin-coating speeds. The results show that the higher the spin-coating speed, the thinner the PI layer formed, and the shorter the required oxygen plasma treatment time. Figure 6b–e display the SEM images of the mask structures fabricated at different speeds. As can be seen in the figure, the minimum height of the nanofibers is about 1 μm. It is known that the mask is consumed during the etching process. Therefore, nanocone structures with different dimensions can be fabricated by adjusting parameters such as the height of the nanofibers.

**Figure 7:**
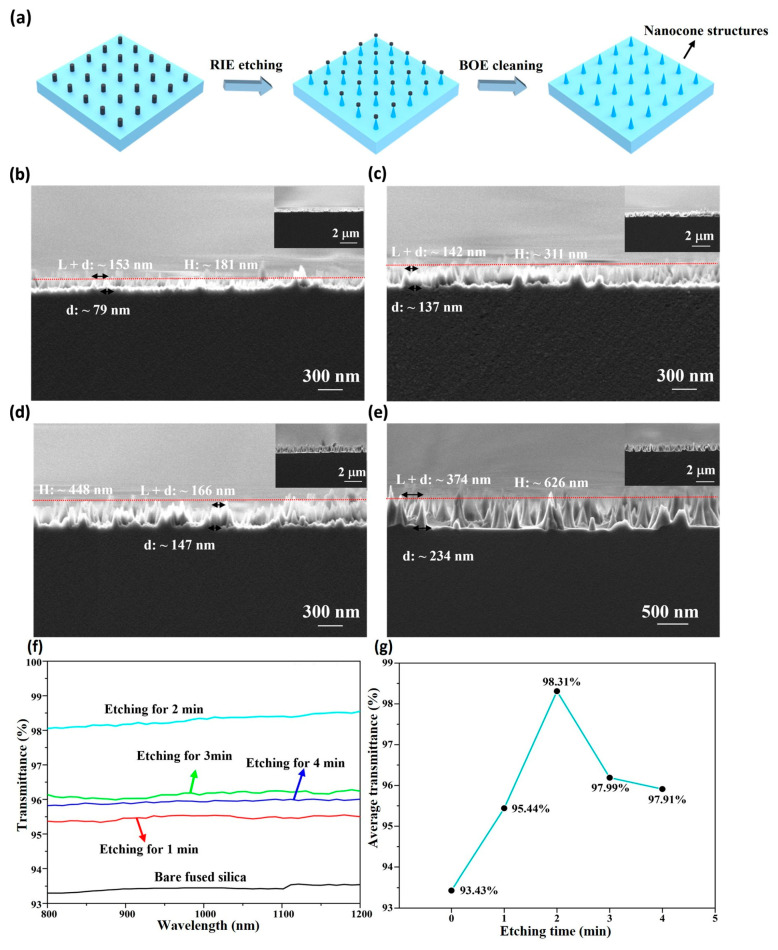
(**a**) Nanocone structure fabrication process. (**b**–**e**) SEM images of nanocones formed via RIE with etching times of (**b**) 1 min, (**c**) 2 min, (**d**) 3 min, and (**e**) 4 min (the red dash line indicates the height). (**f**) Variation in transmittance with wavelength at different etching times. (**g**) Variation in average transmittance with etching time.

After fabricating the nanofiber mask, the nanocone structure was prepared via RIE, and the process flow is illustrated in Figure 7a. Using the nanofibers as a mask, the fused silica substrate was etched with gases such as sulfur hexafluoride to form a composite structure of nanofibers and nanocones. Subsequently, the residual nanofiber mask was removed using BOE. After the fabrication of one side was completed, PI photoresist was spin-coated onto the reverse side of the fused silica substrate. Following oxygen plasma treatment, RIE, and BOE cleaning, the double-sided nanocone structure on the fused silica substrate was finally obtained. We further investigated the influence of different etching durations on the nanocone structure. The relevant parameters are listed in Table 1, and the corresponding SEM images are shown in Figure 7b–e. The insets of Figure 7b–e present low-magnification SEM images of nanocones, demonstrating that the height of the nanocones is highly uniform across a large region. Otherwise, the height and diameter of the nanocone structure increased significantly with the increase in etching time. Under the four etching conditions, the average heights of the nanocone structures are approximately 181 nm, 311 nm, 448 nm, and 626 nm, respectively. We have labeled the diameters of some nanocones in the figures, which are about 79 nm, 137 nm, 147 nm, and 234 nm. Since a complete cross-section of the nanocones cannot be obtained during SEM characterization by cleaving, only the L + d values of partial nanocones can be acquired, which are approximately 153 nm, 142 nm, 166 nm, and 374 nm, respectively. Therefore, the duty cycles of the prepared nanocone structures under the four etching conditions are 0.52, 0.96, 0.89, and 0.63, respectively. After comprehensive comparison of the above dimensions, the nanocone structure prepared under the 2 min etching time matches the optimal structural dimensions from the simulation calculations best. Figure 7f presents the transmittance of the nanocone structures obtained at different etching times. When etched for 1 min, the cone structure was too short in height to achieve a satisfactory antireflection effect. When etched for 2 min, the height of the nanocone structure exceeded 300 nm, and it exhibited excellent optical performance in the 800–1200 nm wavelength band, with a transmittance of 98.31%, representing a 4.88% improvement compared to that of the unprocessed fused silica. Conversely, the transmittance decreased with a further increase in etching time. However, the transmittance of fused silica with the nanocone structure was higher than that of the bare fused silica, which also demonstrates the antireflection effect of the nanocone structure. We therefore finally selected 2 min as the optimal etching time. Figure 7g shows the variation in the average transmittance of fused silica with increasing nanocone height. When the cone height is too low, the graded region is insufficient to realize a complete refractive index transition. When the cone height is excessively high, the aspect ratio of the nanocones becomes overly large, leading to the failure of local effective refractive index grading and matching, which further intensifies surface scattering and therefore results in decreased transmittance. Although the fabricated nanocone structures cannot precisely achieve a single optimal size, by adjusting the etching conditions, nanocones that match the optimal simulated dimensions as closely as possible can be obtained, resulting in a relatively high transmittance.

**Figure 8:**
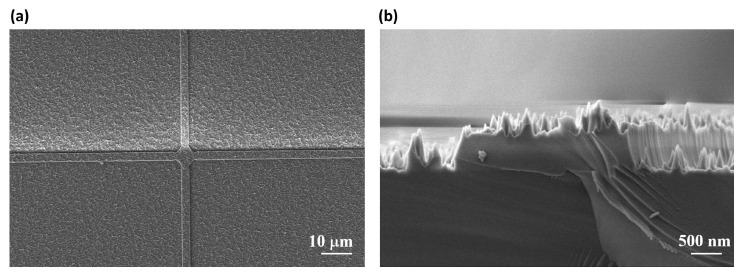
SEM images of the armor-protected nanocone structures. (**a**) Top view at low magnification. (**b**) Side view at high magnification.

After the three critical steps of protective armor construction, nanofiber mask preparation, and final nanocone structure etching, the armor-protected nanocone structure meeting our design requirements was successfully fabricated (protective armor line length of approximately 1000 μm, line width of approximately 2.5 μm, nanocone height greater than 300 nm, and diameter less than 550.96 nm). Throughout the manufacturing process, the preparation of nanofibers and nanocone structures relies heavily on highly controllable RIE parameters, such as precisely adjusted gas composition, pressure, and power. We regulated these parameters to ensure that the desired H, d, and duty cycle were achieved. Figure 8a shows a low-magnification top-view SEM image of the armor-protected nanocone structures, in which the square micron-scale protective framework can be clearly observed. Figure 8b presents a high-magnification detailed view of the protective armor, revealing that nanocone structures are also formed on top of the protective framework. In addition, a large number of nanocones are distributed alongside the sidewalls of the protective armor, with heights lower than that of the framework. When the structure is subjected to surface abrasion, the nanocones inside the framework remain intact and undamaged.

## 5. Wear Testing

**Figure 9:**
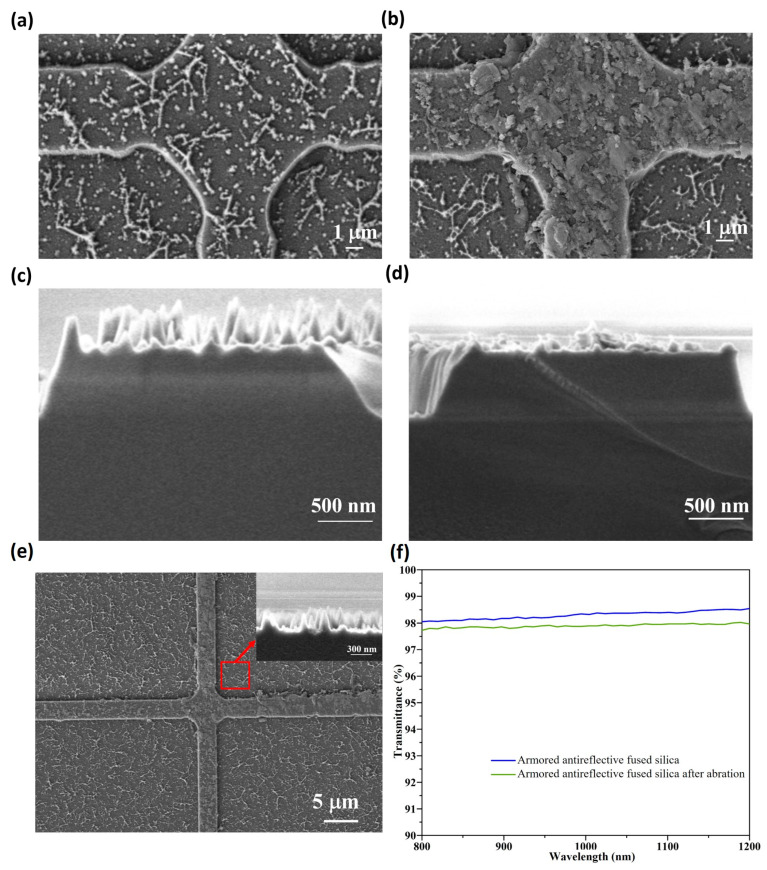
(**a**–**d**) Comparative SEM images of the nanocone structure on the wall surface before and after abrasion. (**e**) Overall SEM image of the nanocone structure protected by micron armor after abrasion. (**f**) Comparison of transmittance of armored antireflective fused silica before and after abrasion.

After fabricating the armor-protected nanocone structures, we characterized their wear resistance. The as-prepared structure was subjected to friction testing using a friction and wear testing machine. The morphological comparison before and after friction (see Figure 9a–d) reveals that the nanocone structures on top of the protective armor were damaged due to the friction. The overall SEM image after friction (see Figure 9e and inset) shows that the nanocone structures inside the protected region remained intact with almost no damage. Therefore, we measured the transmittance of the double-sided armor-protected nanocone structures before and after the friction test, as shown in Figure 9f. The results indicate that the transmittance decreased slightly, but not significantly, after the friction test. In the wavelength range of 800–1200 nm, the transmittance remained at approximately 97.85%. This indicates that the armor-protected nanocone structures can effectively resist external abrasion and maintain excellent antireflection performance.

## 6. Conclusions

In this study, a wear-resistant and antireflective structure based on a double-sided nanocone structure protected by micron armor was successfully designed and fabricated. To minimize the impact of the square-shaped armor structure on optical transmittance while ensuring satisfactory mechanical stability, parameter simulations were carried out. Subsequently, simulation calculations were performed on the height, diameter, and duty cycle of the nanocones, leading to the determination of the following optimal design parameters: diameter < 550.96 nm, height > 300 nm, and duty cycle of approximately 1. For the fabrication of the armor-protected nanocone structures, a hybrid process combining photolithography and RIE was adopted. Specifically, the micron-scale protective armor structure was fabricated first. Thereafter, nanofibers formed by bombarding PI photoresist with oxygen plasma were utilized as an etching mask to prepare nanocone structures with varying dimensions. Ultimately, the double-sided nanocone structure etched for 2 min achieved the highest transmittance of 98.31% in the near-infrared band of 800–1200 nm, which represents a 4.88% improvement compared with bare fused silica. This double-sided nanocone structure exhibits excellent broadband antireflective performance, thereby providing a novel approach for enhancing the performance of optical systems. To assess the wear resistance of the structure, friction tests were conducted on the armor-protected nanocone structures. After reciprocal friction under a pressure of 10 MPa, the nanocone structure protected by the micron armor showed negligible wear, with a transmittance of 97.85% and only a 0.46% decrease from the pre-wear transmittance, thus demonstrating exceptional wear resistance. Owing to its superior anti-reflective and wear-resistant properties, the armor-protected nanocone structure has outstanding potential in broadband anti-reflection applications in optical systems operating in outdoor wear environments.

## Figures and Tables

**Table 1:** Samples etched for different time periods.

Sample	Etching Time/min	H/nm	D/nm	L + d/nm
01	1	~181	30–130	30–200
02	2	~311	100–280	100–290
03	3	~448	100–300	100–325
04	4	~626	200–450	200–625

## Data Availability

The original contributions presented in this study are included in the article. Further inquiries can be directed to the corresponding author.

## References

[1] Forberich K., Dennler G., Scharber M.C., Hingerl K., Fromherz T., Brabec C.J. (2008). Performance improvement of organic solar cells with moth eye anti-reflection coating. Thin Solid Film..

[2] Lee C., Bae S.Y., Mobasser S., Manohara H. (2005). A novel silicon nanotips antireflection surface for the micro sun sensor. Nano Lett..

[3] Park G.C., Song Y.M., Ha J.H., Lee Y.T. (2011). Broadband antireflective glasses with subwavelength structures using randomly distributed Ag nanoparticles. J. Nanosci. Nanotechnol..

[4] Yanagishita T., Nishio K., Masuda H. (2009). Anti-reflection structures on lenses by nanoimprinting using ordered anodic porous alumina. Appl. Phys. Express.

[5] Zang Z., Mukai K., Navaretti P., Duelk M., Velez C., Hamamoto K. (2012). Thermal resistance reduction in high power superluminescent diodes by using active multi-mode interferometer. Appl. Phys. Lett..

[6] Quijada M.A., Del Hoyo J.G., de Marcos L.V.R., Wollack E.J., Batkis M.F., Lewis D.M., Rydalch T.D., Allred D.D. (2024). Enhanced far ultra-violet optical properties of physical vapor deposited aluminum mirrors through fluorination. Proceedings of the Space Telescopes and Instrumentation 2024: Ultraviolet to Gamma Ray.

[7] Firoozifar S., Behjat A., Kadivar E., Ghorashi S., Zarandi M.B. (2011). A study of the optical properties and adhesion of zinc sulfide anti-reflection thin film coated on a germanium substrate. Appl. Surf. Sci..

[8] Oh J.M., Nasir M., Ryu B., Yun H.J., Choi C.J., Bae J.S., Park H.J. (2022). Anomalous optoelectric properties of an ultrathin ruthenium film with a surface oxide layer for flexible transparent conducting electrodes. Adv. Funct. Mater..

[9] Moghadam R.Z., Ahmadvand H. (2021). Optical and Mechanical Properties of ZnS/Ge0.1C0.9 Antireflection Coating on Ge Substrate. Iran. J. Sci. Technol. Trans. Sci..

[10] Leo S.Y., Zhang Y., Jiang J., Lin N., Jiang P., Taylor C. (2024). Reconfigurable antireflection coatings enabled by PDMS oligomer infusion in templated nanoporous polymer films. ACS Appl. Mater. Interfaces.

[11] Xi J.Q., Schubert M.F., Kim J.K., Schubert E.F., Chen M., Lin S.Y., Liu W., Smart J.A. (2007). Optical thin-film materials with low refractive index for broadband elimination of Fresnel reflection. Nat. Photon..

[12] Kikuta H., Toyota H., Yu W. (2003). Optical elements with subwavelength structured surfaces. Opt. Rev..

[13] Chattopadhyay S., Huang Y.F., Jen Y.J., Ganguly A., Chen K.H., Chen L.C. (2010). Anti-reflecting and photonic nanostructures. Mater. Sci. Eng. R Rep..

[14] Ye X., Huang J., Geng F., Liu H., Sun L., Yan L., Jiang X., Wu W., Zheng W. (2016). High power laser antireflection subwavelength grating on fused silica by colloidal lithography. J. Phys. D Appl. Phys..

[15] Ye X., Jiang X.-D., Huang J., Sun L.-X., Geng F., Yi Z., Zu X.-T., Wu W.-D., Zheng W. (2016). Subwavelength structures for high power laser antireflection application on fused silica by one-step reactive ion etching. Opt. Laser Eng..

[16] Toyota H., Takahara K., Okano M., Yotsuya T., Kikuta H. (2001). Fabrication of microcone array for antireflection structured surface using metal dotted pattern. Jpn. J. Appl. Phys..

[17] Mori T., Hasegawa K., Hatano T., Kasa H., Kintaka K., Nishii J. (2008). Surface-relief gratings with high spatial frequency fabricated using direct glass imprinting process. Opt. Lett..

[18] Zhao L., Wang Z., Zhang J., Cao L., Li L., Yue Y., Li D. (2015). Antireflection silicon structures with hydrophobic property fabricated by three-beam laser interference. Appl. Surf. Sci..

[19] Zhang Z., Wang Z., Wang D., Ding Y.-j. (2014). Periodic antireflection surface structure fabricated on silicon by four-beam laser interference lithography. J. Laser Appl..

[20] Leem J.W., Song Y.M., Yu J.S. (2013). Biomimetic artificial Si compound eye surface structures with broadband and wide-angle antireflection properties for Si-based optoelectronic applications. Nanoscale.

[21] Gao X., Yan X., Yao X., Xu L., Zhang K., Zhang J., Yang B., Jiang L. (2007). The dry-style antifogging properties of mosquito compound eyes and artificial analogues prepared by soft lithography. Adv. Mater..

[22] Kanamori Y., Okochi M., Hane K. (2013). Fabrication of antireflection subwavelength gratings at the tips of optical fibers using UV nanoimprint lithography. Opt. Express.

[23] Leem J.W., Kim S., Lee S.H., Rogers J.A., Kim E., Yu J.S. (2014). Efficiency enhancement of organic solar cells using hydrophobic antireflective inverted moth-eye nanopatterned PDMS films. Adv. Energy Mater..

[24] Ge W., Xing C., Veiko V., Li Z. (2019). All-optical, self-focused laser beam array for parallel laser surface processing. Opt. Express.

[25] Salter P.S., Booth M.J. (2019). Adaptive optics in laser processing. Light Sci. Appl..

[26] Xu K., Hu J., Wang M., Cheng G.J., Xu S. (2022). Armored nanocones engraved by selective laser doping enhanced plasma etching for robust supertransmissivity. ACS Appl. Mater. Interfaces.

[27] Xu Z., Zhang Q., Yang Y., Wu H., Xie Y., Yang Y., Feng P., Hou J., Kong L., Tu J. (2024). Hydrophobic antireflective coatings based on the synergistic effect of hollow and solid silica for application in photovoltaic modules. Colloids Surf. A Physicochem. Eng. Asp..

[28] Zhang J., Ai L., Lin S., Lan P., Lu Y., Dai N., Tan R., Fan B., Song W. (2019). Preparation of humidity, abrasion, and dust resistant antireflection coatings for photovoltaic modules via dual precursor modification and hybridization of hollow silica nanospheres. Sol. Energy Mater. Sol. Cells.

[29] Wang D., Sun Q., Hokkanen M.J., Zhang C., Lin F.-Y., Liu Q., Zhu S.-P., Zhou T., Chang Q., He B. (2020). Design of robust superhydrophobic surfaces. Nature.

[30] Li M., Shi M., Wang B., Zhang C., Yang S., Yang Y., Zhou N., Guo X., Chen D., Li S. (2021). Quasi-Ordered Nanoforests with Hybrid Plasmon Resonances for Broadband Absorption and Photodetection. Adv. Funct. Mater..

